# Elucidating the Foundations of Statistical Inference with 2 x 2 Tables

**DOI:** 10.1371/journal.pone.0121263

**Published:** 2015-04-07

**Authors:** Leena Choi, Jeffrey D. Blume, William D. Dupont

**Affiliations:** Department of Biostatistics, Vanderbilt University School of Medicine, Nashville, TN, USA; University of New South Wales, AUSTRALIA

## Abstract

To many, the foundations of statistical inference are cryptic and irrelevant to routine statistical practice. The analysis of 2 x 2 contingency tables, omnipresent in the scientific literature, is a case in point. Fisher's exact test is routinely used even though it has been fraught with controversy for over 70 years. The problem, not widely acknowledged, is that several different p-values can be associated with a single table, making scientific inference inconsistent. The root cause of this controversy lies in the table's origins and the manner in which nuisance parameters are eliminated. However, fundamental statistical principles (e.g., sufficiency, ancillarity, conditionality, and likelihood) can shed light on the controversy and guide our approach in using this test. In this paper, we use these fundamental principles to show how much information is lost when the tables origins are ignored and when various approaches are used to eliminate unknown nuisance parameters. We present novel likelihood contours to aid in the visualization of information loss and show that the information loss is often virtually non-existent. We find that problems arising from the discreteness of the sample space are exacerbated by p-value-based inference. Accordingly, methods that are less sensitive to this discreteness - likelihood ratios, posterior probabilities and mid-p-values - lead to more consistent inferences.

## Introduction

To many, the foundations of statistical inference are cryptic and irrelevant to routine statistical practice. The analysis of 2 × 2 contingency tables, ubiquitous in the scientific literature, is a case in point. A problem, not widely acknowledged, is that several different p-values can be associated with a single table, making scientific inference inconsistent. The analysis of 2 × 2 contingency tables has generated controversy and dispute for more than a half-century in the statistical literature, so perhaps ‘deceptively simple’ would be a better description. For an illustration, consider the data from an example in the right panel of Table 1. Many p-values, including that from Fisher’s exact test, are associated with this one table despite the fact that they all appear to test the same null hypothesis. Table 2 shows these p-values, which range in magnitude and may lead different conclusions. As such, this controversy is often viewed—too simplistically—as a problem of selecting the ‘right p-value’.

**Table 1:** Left: notation used in this paper for the 2 × 2 contingency table; Right: an example data of the 2 × 2 contingency table, which are also presented in Fig. 2 and Example 2 in Fig. 5.

	Success	Failure	Total		Success	Failure	Total
Treatment 1	*y*_1_	*n*_1_−*y*_1_	*n*_1_	Treatment 1	1	9	10
Treatment 2	*y*_2_	*n*_2_−*y*_2_	*n*_2_	Treatment 2	5	5	10
Total	*y*_+_	*n*_+_−*y*_+_	*n*_+_	Total	6	14	20

**Table 2:** P-values obtained from the analysis of example data in Table 1 using several methods.

Analysis method		p-value
Pearson *χ* ^2^ test	without Yates’ continuity correction	0.0509
	with Yates’ continuity correction	0.1432
Fisher’s exact test	two-sided	0.1409
	one-sided	0.0704
*mid*-p-value		0.0758
LR* test		0.0494

* The likelihood ratio (LR) test based on the Equation (9).

What is less known, however, is that the roots of this controversy lie in the table’s origins and the manner in which (hidden) nuisance parameters are eliminated. That is, the roots of the controversy boil down to choosing an appropriate probability model or likelihood function. One needs to specify a probability model, eliminate unknown nuisance parameters, and then interpret the likelihood function (either directly or via some tool such as a p-value). How this is done matters because it leads to different inferences. Intuition alone is not sufficient to guide this activity; inference should be guided by the key foundational principles of sufficiency, ancillarity, conditionality, and likelihood. Other principles such as the Likelihood Principle, the Law of Likelihood, and the Repeated Sampling Principle guide the interpretation of the chosen working likelihood function. Apparent conflict between these principles is the root cause of more familiar queries: “Which is the right p-value?” or “Is the p-value the right tool for reporting the strength of the evidence in the data?” Many statisticians and consumers of statistical methods are in the habit of choosing an approach based on ad-hoc criteria, past experience, or popular trends.

2 × 2 tables have a high profile in the scientific literature as they are among the most common practical applications of basic statistics. The seemingly simple model upon which they are based can be used to illustrate the key principles that apply to all of statistical inference, but that are seldom discussed and often assumed inaccessible. Our goal here is to revisit these principles in the context of 2 × 2 tables and use those principles to show how much information is lost when the tables origins are ignored and when various approaches are used to eliminate the nuisance parameter. We present novel likelihood contours to aid in the visualization of information loss. We show that it is the discreteness of the sample space that is most problematic, and this discreteness is exacerbated when the statistical evidence is summarized with a p-value derived by model conditioning. To get around this difficulty, while maintaining consistency with inferential principles, we suggest summarizing the strength of statistical evidence with a likelihood ratio. Here, the measure of evidence is how much better one hypothesis predicts the observed data than another. Likelihood ratios are less affected by discreteness in the sample space and they provide a reliable sense of the strength of evidence in data. If one absolutely cannot stray from p-values, we suggest back-calculating the p-value from the correct likelihood ratio, as this leads to inferences that are consistent across many types of data and study designs.

## Methods

### Notation

We will focus on parametric models. Let *X* denote a random variable with the probability density function (*pdf*) or probability mass function (*pmf*), *f*_*X*_(*x*;*θ*), which is indexed with a parameter *θ*. We suppress the random variable subscript on the density, i.e., *f*_*X*_(*x*;*θ*) = *f*(*x*;*θ*), as it is clear from the context which density we are using. Upon observing *X* = *x*, the likelihood function for *θ*, defined up to a proportional constant, is *L*(*θ*;*x*) ∝ *f*(*x*;*θ*).

Our notation for 2 × 2 tables is given in the left panel of Table 1. Here *y*_1_ and *y*_2_ are realizations of random variables *Y*_1_ and *Y*_2_. Depending on the context, sample sizes *n*_1_ and *n*_2_ can be realizations of random variables *N*_1_ and *N*_2_, but they are most often fixed by design. Following our convention, we use *f*(*y*_*i*_; *π*_*i*_,*n*_*i*_) instead of the more precise *f*(*y*_*i*_; *π*_*i*_,*N*_*i*_ = *n*_*i*_), when the meaning is clear from the context.

### Background

Perhaps the most widely applied statistical method in the scientific literature, Fisher’s exact test has elicited enormous controversy over the past 70 years. The controversy over how to report the strength of statistical evidence in 2 × 2 contingency tables has many facets, but it is often over-simplified and cast as a debate about choosing one p-value over another. Less well-known is that many of the criticisms of Fisher’s exact test also apply to Yates’ continuity-corrected chi-square test [1], because it makes the *χ* ^2^ distribution under the null closer to the hypergeometric distribution on which Fishers exact test is based. Readers unfamiliar with the controversy will find ample background in: Conover [2], Yates [3], Haviland [4], and a series of papers [5–8] responding to Berkson [9]. A more recent review is by Agresti [10]. Here we briefly summarize the controversy over Fisher’s exact test (and Yates’ continuity corrected *χ* ^2^ test), which will be discussed in detail in the following sections with some examples as we step through this controversy. We also present three main models for 2 × 2 contingency tables that will provide the basis of specifying the working likelihood.

#### Summary of controversy

Although all issues of the controversy are interconnected, their roots entail three main issues: (1) conditionality; (2) conservatism; and (3) modeling assumptions.

##### Conditionality

The conditionality debate is the most controversial. Fisher’s exact test was derived by conditioning on the observed success total (*y*_+_) in Table 1, and the major concern is about the loss of information due to this conditioning. This loss of information has been discussed by many authors including Kalbfleisch and Sprott [11], Plackett [12], Haber [13], Yates [3], and Zhu and Reid [14]. The opponents of conditioning argue that we cannot condition on the observed success total since it is not a perfect ancillary statistic, or that the tests modeling assumptions are false (see below). Proponents argue that per the Conditionality Principle, the inference should be made by conditioning on the observed success totals which are approximately ancillary with little loss of information; hence this group is supportive of Fisher’s exact test.

##### Conservatism

Conservatism means that the actual probability of rejecting the null hypothesis is less than the nominal level. Fisher’s exact test is indeed conservative, and this is easily verified by simulation. Conover [2], D’Agostino *et al.*[15], Grizzle [16], Starmer *et al.*[17] and Plackett [18] discuss this in detail. In response to this criticism, Tocher [19] proposed a randomization test to improve Fisher’s exact test so that the significance level can be precisely attained. Although this test is a most powerful test, it has been rarely implemented in practice. A similar idea is found in the *mid*-p-value suggested by Lancaster [20], which is recommended by several authors including Stone [21] and Upton [22]. The conservatism of Fisher’s exact test is mainly due to the discreteness of the test statistic. This discreteness impacts conditional tests, such as Fisher’s exact test, much more than unconditional tests [23]. We later illustrate how the sample space can be dramatically reduced by conditioning due to discreteness. Dupont [24] shows that very minor perturbations of the tables can lead to substantial changes in the p-value. His solution was to double the one-sided p-value [3, 24].

##### Modeling assumptions

The argument over the correct model for 2 × 2 tables concerns the margins of Table 1: one fixed margin (i.e., the sample size, *n*_1_ and *n*_2_, fixed) versus two fixed margins models (i.e., the sample size and the total number of successes, *y*_+_, fixed). Pearson [25] and Kempthorne [8] discussed three models based on the origin of the data: (1) zero margins fixed; (2) one margin fixed; (3) two margins fixed. The corresponding models use multinomial, two independent binomials and hypergeometric distributions, respectively, as specified below. They argued that Fisher’s exact test is appropriate only for data where the two margins are fixed by the study design. On the other hand, Barnard [5, 26] argued that the last two models should be distinguished based on whether there are two underlying populations who have their own constant probabilities *π*_1_ and *π*_2_; for this case, the two independent binomials are the correct model since the two binomials have their own parameters, *π*_1_ and *π*_2_. Otherwise, the two margins fixed model (i.e., hypergeometric distribution indexed with a single parameter) is correct as is Fisher’s exact test. The zero margin fixed model has been rarely an issue since the sample size margin is usually agreed to be fixed. The other margin for the total number of success (called as “the incidence margin” by Cormack and Mantel [27]) has been the focus of controversy. From a different standpoint, Greenland [28] also argued in favor of Fisher’s exact test.

### Three main models

Two-by-two contingency tables arise in several ways, and their genesis often suggests a natural model. For example, 2 × 2 tables can be generated by studies where two groups of subjects (those exposed to some risk factor and those not exposed) are followed to determine the incidence of a certain disease in each group. For these data, *y*_1_ and *y*_2_ can be thought of as realized counts of independent random variables *Y*_1_ and *Y*_2_ with the total number of subjects in each group, *n*_1_ and *n*_2_, fixed by design. A natural statistical model for these data is *Y*_1_ ∼ Binomial (*n*_1_, *π*_1_) and *Y*_2_ ∼ Binomial (*n*_2_, *π*_2_), which yields the following joint *pmf*

$$\begin{array}{ccc} f(y_{1},y_{2};\;\pi_{1},\pi_{2},n_{1},n_{2}) & = & f\left(y_{1};\;\pi_{1},n_{1}\right)f\left(y_{2};\;\pi_{2},n_{2}\right)\\ & = & \left[\left(\begin{array}{c} n_{1}\\ y_{1} \end{array}\right)\pi_{1}^{y_{1}}{\left(1-\pi_{1}\right)}^{\left(n_{1}-y_{1}\right)}\right]\left[\left(\begin{array}{c} n_{2}\\ y_{2} \end{array}\right)\pi_{2}^{y_{2}}{\left(1-\pi_{2}\right)}^{\left(n_{2}-y_{2}\right)}\right]. \end{array}$$ (1)

Typically, one tests the null hypothesis that *π*_1_ = *π*_2_, which is equivalent, under the null hypothesis, to testing that the odds ratio, $\frac{\pi_{1}(1-\pi_{2})}{\pi_{2}(1-\pi_{1})}=1$, or the log odds ratio $\psi =\log\left\{\frac{\pi_{1}(1-\pi_{2})}{\pi_{2}(1-\pi_{1})}\right\}=0$. The choice of a parameter of interest is important, and we focus on inference about the odds ratio or the log odds ratio because it is the most prevalent in the applied literature.

In contrast, studies may fix the total number of subjects in each group, *n*_1_ and *n*_2_ (or equivalently, *n*_1_ and the total number of subjects, *n*_+_ = *n*_1_ + *n*_2_), and the total number of success, *y*_+_, due to limited resources or practical constraints. For example, studies with a fixed number of participants are followed until a set number of total events are observed. In this case, *Y*_1_ ∼ Hypergeometric (*y*_+_, *n*_1_, *n*_2_) under the null hypothesis that *ψ* = 0. This is the *pmf* underlying Fisher’s exact test:

$$\begin{array}{c} f\left(y_{1}|y_{+};\psi ,n_{1},n_{2}\right)=\left.\left(\begin{array}{c} n_{1}\\ y_{1} \end{array}\right)\left(\begin{array}{c} n_{2}\\ y_{+}-y_{1} \end{array}\right)/\left(\begin{array}{c} n_{+}\\ y_{+} \end{array}\right).\right. \end{array}$$ (2)

Finally, it is possible that no margin totals are fixed in advance. Let *X* and *Y* denote categorical response variables with two categories each, which are obtained from a subject randomly chosen from some population. The responses (*X*,*Y*) can be cross-classified in a 2 × 2 table with cell counts at the *i*th row and the *j*th column, *m*_*ij*_. If we assume the cell counts, *m*_*ij*_, as independent Poisson random variables with parameters *μ*_*ij*_, then the joint *pmf* is the product of the Poisson probabilities for the *ij* cell counts

$$\begin{array}{c} \prod_{i=1}^{2}\prod_{j=1}^{2}\frac{\exp\left(-\mu_{ij}\right)\mu_{ij}^{m_{ij}}}{m_{ij}!}. \end{array}$$

If the table is conditioned on either the row or column margin, say the row totals, *m*_1+_ = *m*_11_ + *m*_12_ and *m*_2+_ = *m*_21_ + *m*_22_, the conditional *pmf* for the cell counts, *m*_*ij*_, given the row totals is

$$\begin{array}{ccc} & & f(m_{11},m_{12},m_{21},m_{22}|m_{1+},m_{2+};\mu_{11},\mu_{12},\mu_{21},\mu_{22})\\ & & \;=\frac{m_{1+}!}{m_{11}!m_{12}!}{\left(\frac{\mu_{11}}{\mu_{11}+\mu_{12}}\right)}^{m_{11}}{\left(\frac{\mu_{12}}{\mu_{11}+\mu_{12}}\right)}^{m_{12}}\frac{m_{2+}!}{m_{21}!m_{22}!}{\left(\frac{\mu_{21}}{\mu_{21}+\mu_{22}}\right)}^{m_{21}}{\left(\frac{\mu_{22}}{\mu_{21}+\mu_{22}}\right)}^{m_{22}}. \end{array}$$ (3)

Letting *m*_11_ = *y*_1_, *m*_21_ = *y*_2_, *m*_1+_ = *n*_1_, *m*_2+_ = *n*_2_, $\pi_{1}=\left(\frac{\mu_{11}}{\mu_{11}+\mu_{12}}\right)$ and $\pi_{2}=\left(\frac{\mu_{21}}{\mu_{21}+\mu_{22}}\right)$, the *pmf* based on two independent binomials (1) can be recovered.

Thus, there is a close relationship between Equations (1), (2), and (3), which we will detail in the next section. The controversy surrounding 2 × 2 tables arises, in part, because different modeling strategies from identical tables can lead to different inferences.

### Principles of inference

Suppose the data in Table 1 represent a sample drawn from some target population of interest (say, all patients with some disease). The *pmf* provides the link between what we know (the data) and what we would like to know (the values of the parameters in the target population). Does Table 1 represent statistical evidence that the first treatment generates more successes than the second in the target population? Naturally, the answer to this question depends on how we construct the probability model which, in turn, facilitates a comparison of the two success rates.

Consider the two independent binomial models (1). If *λ* = log{*π*_2_/(1−*π*_2_)}, then *π*_1_ = exp(*ψ* + *λ*)/[1 + exp(*ψ* + *λ*)] and *π*_2_ = exp(*λ*)/[1 + exp(*λ*)]. Substituting these expressions for *π*_1_ and *π*_2_ into Equation (1) yields

$$\begin{array}{c} f\left(y_{1},y_{2};\psi ,\lambda ,n_{1},n_{2}\right)=\left(\begin{array}{c} n_{1}\\ y_{1} \end{array}\right)\left(\begin{array}{c} n_{2}\\ y_{2} \end{array}\right)\exp\left(\psi y_{1}\right)\exp\left(\lambda y_{+}\right){\left[1+\exp(\psi +\lambda )\right]}^{-n_{1}}{\left[1+\exp(\lambda )\right]}^{-n_{2}}. \end{array}$$ (4)

The obvious problem here is that in order to use this model, we need to know *λ*, even though it is not of primary interest. In this context, *λ* is a nuisance parameter. There are several ways to deal with nuisance parameters, and an important aspect of the debate about 2 × 2 tables concerns how we do this. Traditional methods such as conditioning on sufficient and ancillary statistics to eliminate nuisance parameters are motivated by foundational principles. A formal discussion of these principles is beyond the scope of this paper (c.f., [29–31]), however an intuitive and contextual discussion is illuminating for 2 × 2 tables.

#### Sufficiency Principle

Following Casella and Berger [30] and Reid [31], a statistic *S*(*Y*) is a sufficient statistic for a parameter *θ* if the conditional distribution of *Y* given the value of *S*(*Y*) does not depend on *θ*. Suppose there exists a one-to-one transformation from *Y* to (*S*(*Y*),*B*(*Y*)) such that

$$\begin{array}{c} f(y;\theta )\propto f(s;\theta )f(b|s), \end{array}$$

where the Jacobian of the transformation from *y* to (*s*,*b*) is absorbed in the proportionality constant and the density notation *f*(⋅) indicates the *pdf* or *pmf*. Then, *S*(*Y*) is a sufficient statistic for *θ* because, from a likelihood perspective,

$$\begin{array}{c} L(\theta ;y)\propto L(\theta ;s), \end{array}$$

where the likelihood function depends on the data *y* only through *s*. The Sufficiency Principle asserts that no information about *θ* is lost if we use the marginal *pmf* *f*(*s*;*θ*) or the marginal likelihood function for *s* to make inferences about *θ*.

Sufficiency is used for data reduction. A sufficient statistic contains the same amount of information about the parameter of interest as the original data since the likelihood function based on the sufficient statistic is equal to, up to a proportional constant, the likelihood function based on the entire dataset. Likelihood based inference is therefore preserved. The Sufficiency Principle says that inference based on a sufficient statistic should not be any different from inference based on the data themselves.

#### Ancillarity and Conditionality Principles

Ancillarity is the conceptual opposite of sufficiency in that an ancillary statistic contains no information about the parameter of interest (i.e., its distribution is not a function of this parameter). What do we do with ancillary statistics? Fisher’s conjecture was that we should always condition on them when making inference.

The Conditionality Principle is broader than a directive to condition on ancillary statistics, although that directive is implied. The Conditionality Principle asserts that the statistical evidence about the parameter of interest depends only on the observed data. Experiments and data that could have been observed, but were not, are irrelevant when interpreting the observed data as statistical evidence. The interested reader is referred to Cox [32] for a broad discussion of this principle.

To understand the motivation for this, suppose that we write a grant application to study treatments 1 and 2 in which we propose to set *n*_1_ = *n*_2_ = 100. If we can assume that the probability of funding is unrelated to the magnitude of *ψ*, then the sample size is an ancillary statistic for this parameter. In the current funding climate, there is an all-to-high probability that our grant will not be funded and hence that *n*_1_ = *n*_2_ = 0. However, if our grant is funded, we will condition our inferences about *ψ* on our sample size of 100 patients per treatment group, which we will treat as being fixed. If we are funded, it makes no sense to try to account for our funding chances when making inferences about *ψ*.

As noted in Casella and Berger [30], there are many technical definitions of ancillarity. Reid [31] gives a nice presentation of the notions of sufficiency and ancillarity for models indexed by a single parameter and models that have nuisance parameters. We follow those definitions throughout this section, and recommend her paper to readers who wish to learn more about the roles of conditioning in inference. A statistic *A*(*Y*) is an ancillary statistic for *θ* if there exists a one-to-one transformation from *Y* to (*T*(*Y*),*A*(*Y*)) such that

$$\begin{array}{c} f(y;\theta )\propto f(t|a;\theta )f(a). \end{array}$$

The Ancillarity Principle asserts that if *A*(*Y*) is an ancillary statistic for *θ*, then inference about *θ* should be based on *f*(*t*∣*a*;*θ*), the conditional *pmf* of *t* given *a*. In likelihood terms,

$$\begin{array}{c} L(\theta ;y)\propto L(\theta ;t|a), \end{array}$$

and inference about *θ* is based on the conditional likelihood *L*(*θ*;*t*∣*a*).

This principle is generally less accepted than the idea of using a marginal density or likelihood based on a sufficient statistic. This is probably because ancillary statistics are hard to construct and can be non-unique [29, 31]. In a likelihood sense, *A*(*Y*) = *a* tells us nothing about *θ* since *f*(*a*) does not involve *θ*. Hence, conditioning our inference on *A*(*Y*) = *a* is, at worst, harmless. However, there are situations where conditioning on an ancillary statistic is crucial to achieving sensible inference.

In the common 2 × 2 table setting, the number of subjects in each arm, *n*_1_ and *n*_2_, are considered ancillary statistics because the distribution of the observed number of subjects is assumed free of *π*_1_ and *π*_2_. That is, the number of subjects in each arm is not determined by the underlying probability of success in each arm. This is trivially true in fixed sample size experiments where resources and time determine *n*_1_ and *n*_2_. It would not be true if the trial used adaptive randomization, where participants are more likely to be assigned to the treatment arm that appears, at the time, to be performing better. For the rest of our discussion, we assume that *n*_1_ and *n*_2_ are indeed ancillary statistics, as this is the largely unspoken historical assumption.

For the two-parameter case, *θ* = (*ψ*,*λ*), an ideal situation is when there exists a one-to-one transformation from *Y* to (*S*_1_(*Y*),*S*_2_(*Y*)) such that the model factorizes as:

$$\begin{array}{c} f\left(s_{1},s_{2};\theta \right)\propto f\left(s_{1}|s_{2};\psi \right)f\left(s_{2};\lambda \right). \end{array}$$

Accordingly, we say that *s*_2_ is sufficient for *λ* and ancillary for *ψ*, and inference for *λ* should be based on *f*(*s*_2_;*λ*), the marginal *pmf*, while inference for *ψ* should be based on *f*(*s*_1_∣*s*_2_;*ψ*), the conditional *pmf* of *s*_1_ given *s*_2_.

However, it is more common that we have the following factorization:

$$\begin{array}{c} f\left(y_{1},y_{2};\theta \right)\propto f\left(s_{1}|s_{2};\psi \right)f\left(s_{2};\psi ,\lambda \right), \end{array}$$ (5)

which is in likelihood terms

$$\begin{array}{c} L\left(\theta ;y_{1},y_{2}\right)\propto L\left(\psi ;s_{1}|s_{2}\right)L\left(\psi ,\lambda ;s_{2}\right). \end{array}$$

Now inference about *ψ* and *λ* is no longer easy. Back to the 2 × 2 table, the *pmf* in Equation (4) has two parameters *θ* = (*ψ*,*λ*), and the *pmf* can be factorized as:

$$\begin{array}{ccc} f(y_{1},y_{2};\theta ,n_{1},n_{2}) & \propto & f\left(y_{1}|y_{+};\psi ,n_{1},n_{2}\right)f\left(y_{+};\psi ,\lambda ,n_{1},n_{2}\right),\;\;\text{where} \end{array}$$ (6)

$$\begin{array}{ccc} f(y_{1}|y_{+};\psi ,n_{1},n_{2}) & = & \left.\left(\begin{array}{c} n_{1}\\ y_{1} \end{array}\right)\left(\begin{array}{c} n_{2}\\ y_{+}-y_{1} \end{array}\right)\exp\left(\psi y_{1}\right)/C\left(\psi ,y_{+}\right),\right. \end{array}$$ (7)

$$\begin{array}{ccc} f(y_{+};\psi ,\lambda ,n_{1},n_{2}) & = & \exp\left(\lambda y_{+}\right){\left[1+\exp(\psi +\lambda )\right]}^{-n_{1}}{\left[1+\exp(\lambda )\right]}^{-n_{2}}C\left(\psi ,y_{+}\right), \end{array}$$ (8)

$$\begin{array}{ccc} \text{and} & & \\ C(\psi ,y_{+}) & = & \sum_{u=\max(0,y_{+}-n_{2})}^{\min(n_{1},y_{+})}\left(\begin{array}{c} n_{1}\\ u \end{array}\right)\left(\begin{array}{c} n_{2}\\ y_{+}-u \end{array}\right)\exp\left(\psi u\right). \end{array}$$

This factorization has been discussed by many including Zhu and Reid [14], Reid [31], and McCullagh and Nelder [33]. Notice the similarity in the forms of Equations (5) and (6). The conditional *pmf* in Equation (7), *f*(*y*_1_∣*y*_+_;*ψ*,*n*_1_,*n*_2_), does not depend on *λ*, so it is tempting to use this conditional *pmf* in place of Equation (6), *f*(*y*_1_,*y*_2_;*θ*,*n*_1_,*n*_2_), for inference about *ψ*. The problem is that *y*_+_ is not ancillary for *ψ*, so the remainder in Equation (8), *f*(*y*_+_;*ψ*,*λ*,*n*_1_,*n*_2_), is not free of *ψ* and some information is lost when this remainder is ignored. In short, treating, *y*_+_ as an ancillary statistics when it is not risks loosing information that may or may not be critical to our inferences about *ψ*. If the lost information is negligible, then it is often worth pretending that *y*_+_ is ancillary (and condition on it), since the resulting conditional likelihood is only indexed by the parameter of interest. This allows ‘exact’ inference without resorting to an approximation. Under the null hypothesis that *ψ* = 0, the non-central hypergeometric *pmf* in Equation (7) reduces to the hypergeometric distribution in Equation (2). Fisher’s exact test is based on this conditional distribution. This is why it is called an ‘exact’ test and recommended when the sample size is small or the data are very unbalanced. In these situations, the *χ* ^2^ approximation on which Pearson’s *χ* ^2^ test is based may be poor.

To develop intuition about the extent to which *y*_+_ behaves like an ancillary statistic, consider the following example. Suppose that *n*_1_ = *n*_2_ = 10 and that *y*_+_ = 4. Knowing the value of *y*_+_ tells us that *y*_1_ must take one of the values 0, 1, 2, 3, or 4. This implies that the MLE of the odds ratio, $\exp(\hat{\psi })=[y_{1}/(n_{1}-y_{1})]/[y_{2}/(n_{2}-y_{2})]$ must be 0, 7/27, 1, 27/7, or +∞. The inferential implication is that the evidence about *ψ* contained in *y*_+_ = 4 is equally consistent with odds ratios suggesting that Treatment 1 is inferior to Treatment 2 as it is with odds ratios suggesting that Treatment 1 is superior to Treatment 2. Moreover, as long as *n*_1_ = *n*_2_, then for each possible value of $\exp(\hat{\psi })$ favoring Treatment 1, there is a reciprocal value favoring Treatment 2 (e.g., 7/27 versus 27/7). Hence, *y*_+_ tells us virtually nothing about the true value of *ψ*.

## Results and Discussion

### Information loss when the marginal distribution of *y*_+_ is ignored

The loss of information has been discussed by many authors [3, 11–14]. As discussed above, the success total is not ancillary, and the open question is how much information about *ψ* is contained in the marginal *pmf* *f*(*y*_+_;*ψ*,*λ*,*n*_1_,*n*_2_). Kalbfleisch and Sprott [11] called the conditional likelihood of *ψ* given *y*_+_ an *approximate conditional likelihood*, arguing that there is little information about *ψ* in the marginal *pmf*. For the same reason, *y*_+_ is called an *approximate ancillary statistic* by Little [34].

It is not easy to quantify the amount of information lost with a single numerical summary. So instead, and more intuitively, we visualize the information lost by examining the surface of the marginal likelihood function. For a clearer visualization, we use a subtler reparameterization of the original dual binomial likelihood. Instead of (*π*_1_,*π*_2_) → (*ψ*,*λ*), it is more convenient to use (*π*_1_,*π*_2_) → (*ψ*,λ*), where λ* = (*n*_1_ *π*_1_ + *n*_2_ *π*_2_)/(*n*_1_ + *n*_2_) is the marginal probability of successes among all treated subjects. This latter reparameterization is an orthogonal representation with respect to the expected Fisher’s information matrix [35, 36].

**Fig 1:**
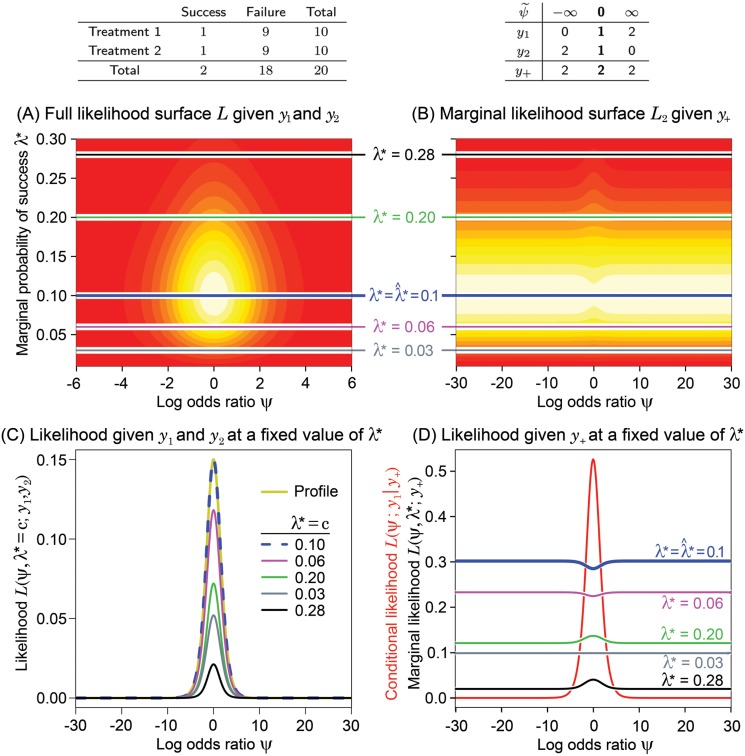
The data are shown on the top left panel. On top right panel, all possible configurations of tables (*y*_1_ and *y*_2_) are listed when only *y*_+_ is known. The corresponding maximum likelihood estimate of the log odds ratio *ψ* for each possible table, denoted as $\tilde{\psi }$, is also shown. The nuisance parameter λ* = (*n*_1_ *π*_1_+*n*_2_ *π*_2_)/(*n*_1_ + *n*_2_) is the marginal probability of success among all treated subjects. (A) Contour plot of the likelihood *L* = *L*(*ψ*,λ*;*y*_1_,*y*_2_), which is the joint likelihood of different values of *ψ* and λ* given the observed values of*y*_1_ and *y*_2_. Lighter colors denote higher values of *L*; (B) Contour plot of the marginal likelihood *L*_2_ = *L*(*ψ*,λ*;*y*_+_) given the success total *y*_+_ as a function of *ψ* andλ*; (C) The likelihood *L* given *y*_1_ and *y*_2_ plotted against *ψ* at five different fixed values ofλ*. The profile likelihood function is also plotted; (D) The marginal likelihood *L*_2_ given *y*_+_ plotted against *ψ* at fixed values of λ*. The conditional likelihood *L*_1_ = *L*(*ψ*;*y*_1_∣*y*_+_) is also plotted in red. These graphs demonstrate that for balanced sample sizes the marginal success total tells us virtually nothing about *ψ*, and hence should be treated as an ancillary statistic.

**Fig 2:**
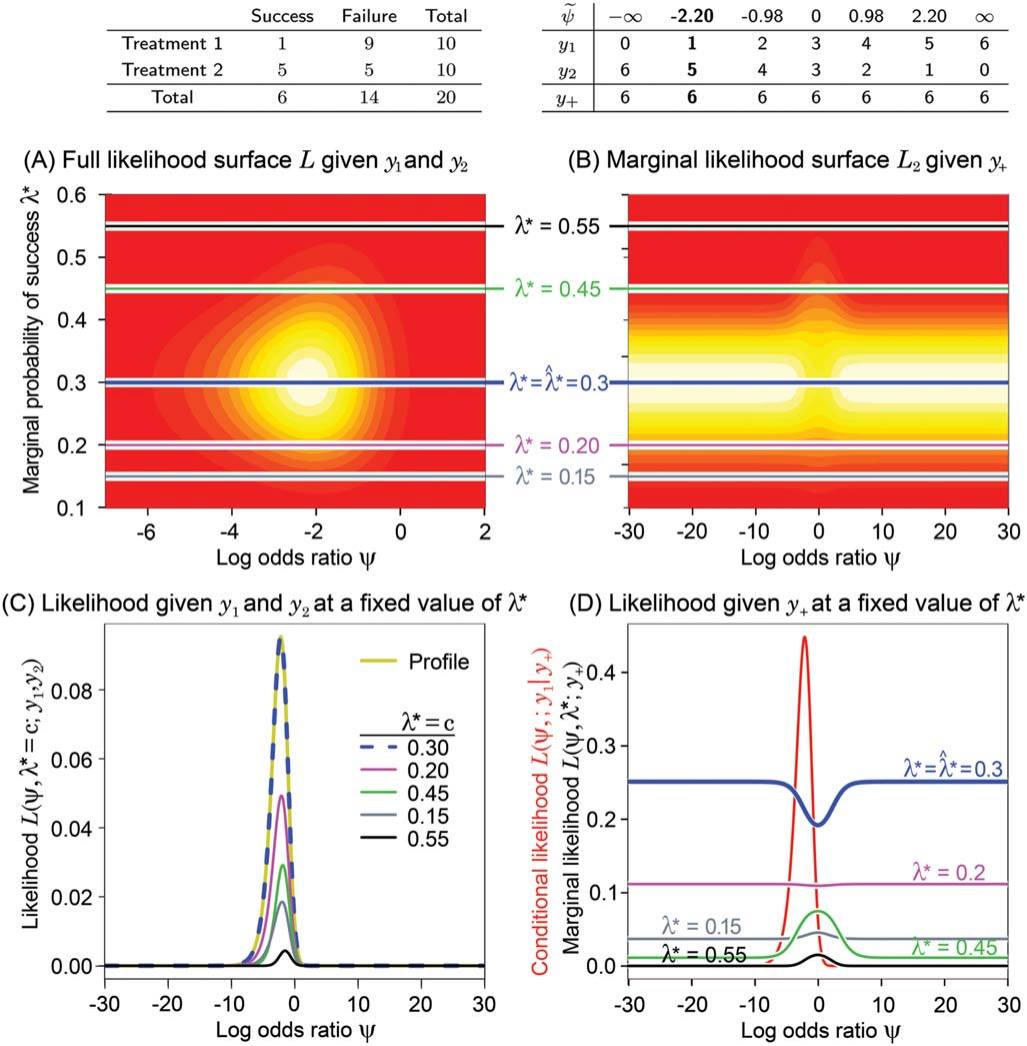
See Fig. 1 for an explanation of these panels. In this example, the sample sizes are the same for both treatments, but the success rates are different.

**Fig 3:**
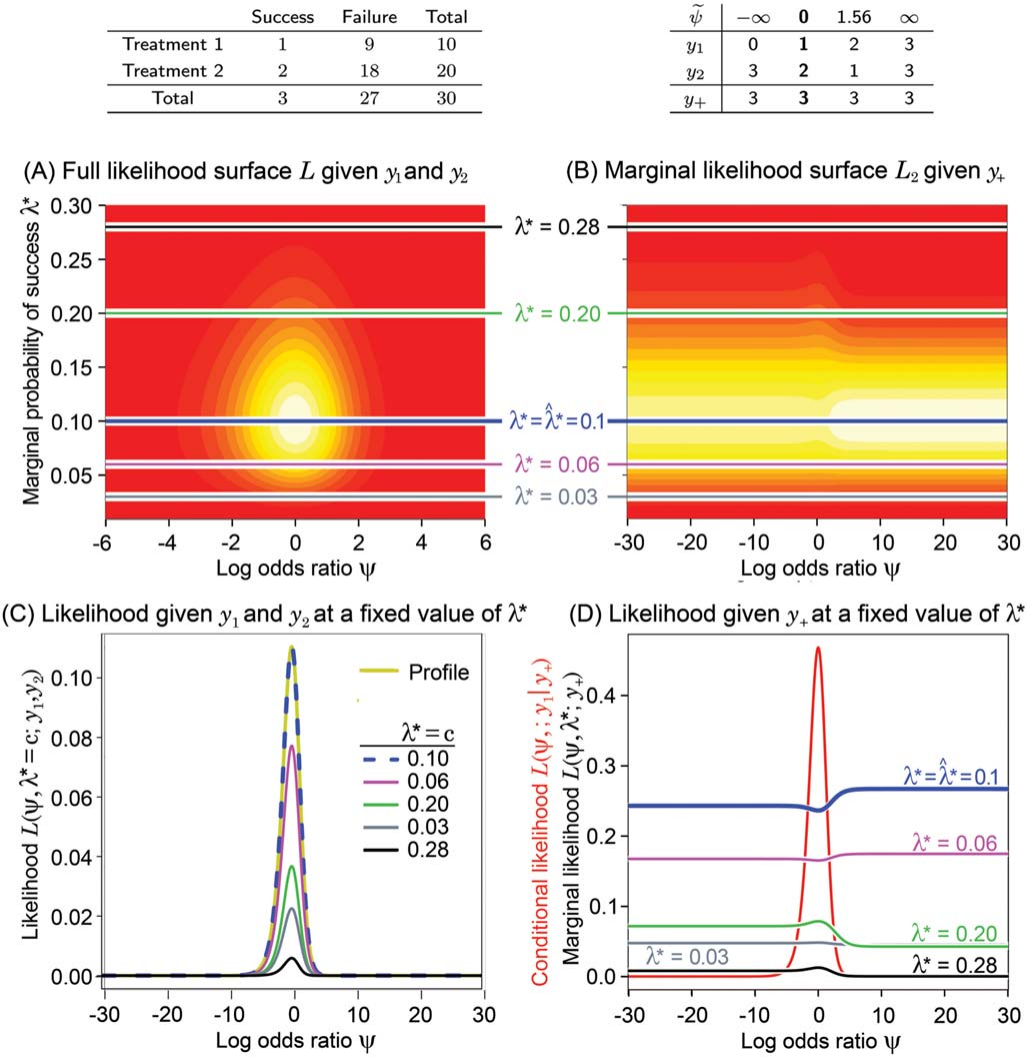
See Fig. 1 for an explanation of these panels. In this example, the treatments have unequal sample sizes. For these tables, the marginal success total still tells us very little about *ψ* although it is slightly more informative than in balanced tables (see also Fig. 4).

**Fig 4:**
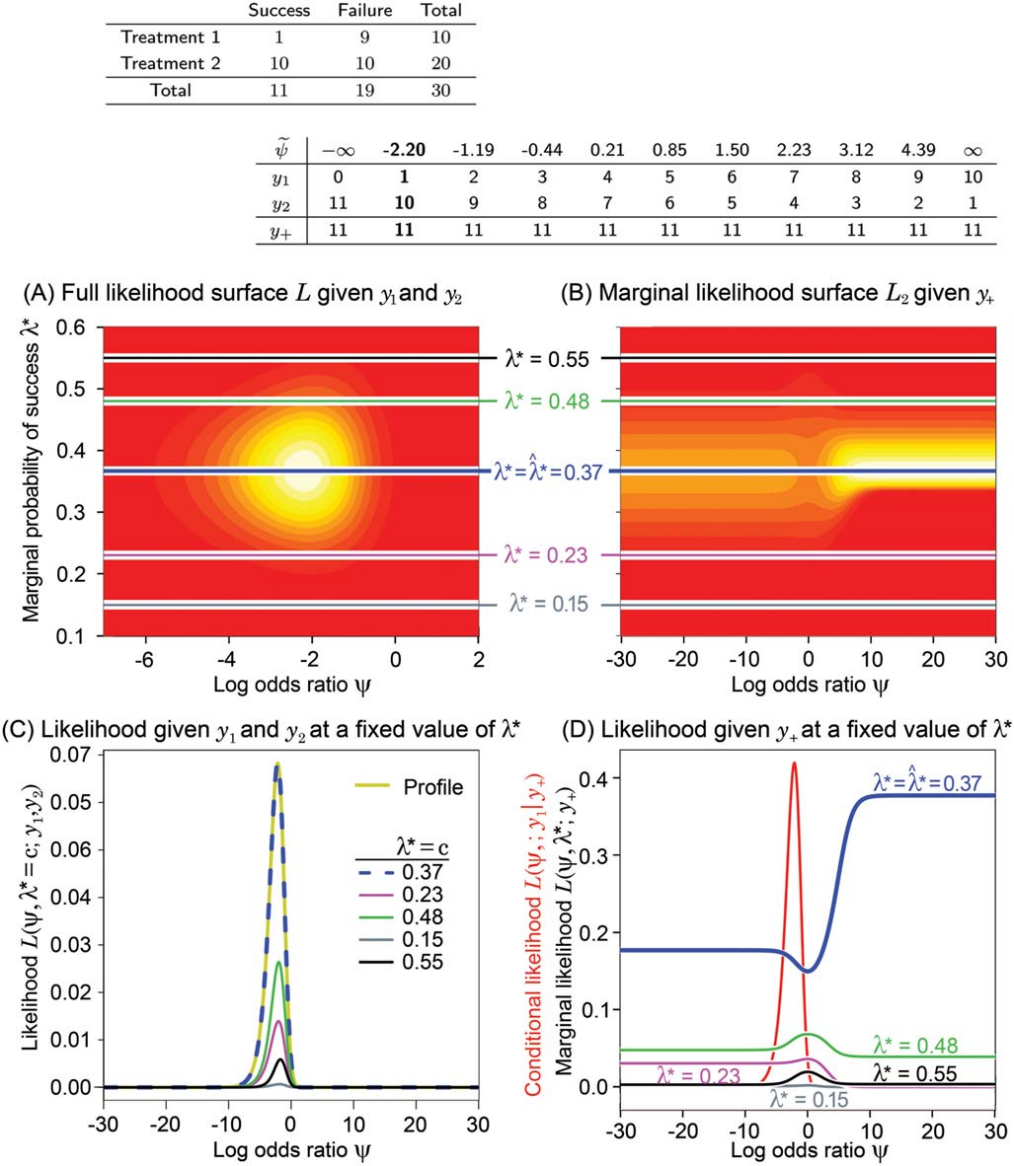
See Fig. 1 for an explanation of these panels. This example has both unequal sample sizes and unequal success rates. It is even more extreme than the example in Fig. 3.

We examined the information about *ψ* contained in *y*_+_ under a wide variety of scenarios, including when the sample sizes are equal, small, large and extremely unbalanced with sparse cells. Fig. 1 and Fig. 2 show examples with equal smaller sample sizes, while Fig. 3 and Fig. 4 show examples with unequal sample sizes. In addition, in Fig. 1 and Fig. 3 the observed success rates are equal while in Fig. 2 and Fig. 4 they are not.

Fig. 1, Fig. 2, Fig. 3 and Fig. 4 have the same layout, but are based on the data shown in the top left panel of each figure (above panel (A)). Denote *L* = *L*(*ψ*,λ*;*y*_1_,*y*_2_), *L*_1_ = *L*(*ψ*;*y*_1_∣*y*_+_), and *L*_2_ = *L*(*ψ*,λ*;*y*_+_). Hence, *L* ∝ *L*_1_ *L*_2_, where *L* is the full likelihood, *L*_1_ is the likelihood given *y*_1_ conditioned on the total number of successes, *y*_+_, and *L*_2_ is the marginal likelihood given *y*_+_. The 2-dimensional surfaces of *L* [panel (A)] and *L*_2_ [panel (B)] are displayed as functions of *ψ* and λ*. The continuum of yellow to red is used to indicate the value of the likelihood function (yellow is higher than red). Wide ranges for the parameter space are intentionally used to increase the utility of this visual examination.

**Fig 5:**
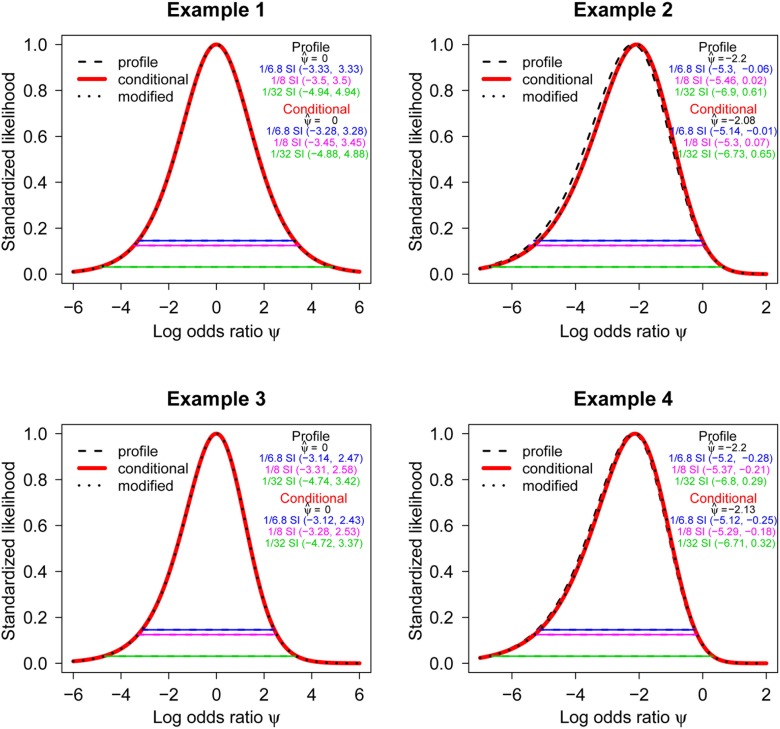
The standardized conditional, modified profile, and profile likelihood functions are depicted for the log odds ratio *ψ* using the data in Fig. 1, Fig. 2, Fig. 3 and Fig. 4. The example numbers in this figure correspond to the examples described in Figs. 1–4. The profile likelihood is represented by a dashed black line, while the conditional and modified profile likelihoods are represented by thick red and black dotted lines, respectively. The horizontal lines represent 1/6.8 (upper), 1/8 (middle) and 1/32 (lower) likelihood support intervals (SIs). The maximum likelihood estimate (MLE) $\hat{\psi }$ of each likelihood was also shown. For normally distributed data, a 1/6.8 SI and a Frequentist 95% confidence interval are identical. Note that the modified profile and conditional likelihoods are indistinguishable for all examples, while the profile and conditional likelihoods are similar for the examples of the null (i.e., *ψ* = 0 in Examples 1 & 3). In these two examples, the profile likelihood is not visible because it is overlain by the conditional likelihood.

Above panel (B), we have displayed in a table all possible configurations of *y*_1_ and *y*_2_, subject to the constraint of the observed *y*_+_. The corresponding MLE of *ψ* for each possible table, denoted as $\tilde{\psi }$, is also shown. The real observed data *y*_1_ and *y*_2_ and observed MLE, $\hat{\psi }$, are in bold text. Panel (D) displays the conditional likelihood *L*_1_ in red along with various cross sections of the marginal likelihood surface, *L*_2_, plotted in panel (B) (values of λ* are shown for reference). In a similar fashion, panel (C) displays the profile likelihood for *ψ* derived from *L*, as well as various cross sections of *L* assuming different fixed values of λ*. In these examples, the profile likelihoods are the same as the estimated likelihoods obtained by plugging $\lambda^{*}={\hat{\lambda }}^{*}$, where ${\hat{\lambda }}^{*}$ is the MLE of λ* (the profile and estimated likelihoods are defined in S1 Appendix). This happens when *ψ* and λ* are orthogonal parameters, as is the case here, but is not true in general. Once the conditional and profile likelihoods are standardized by their maximum, they are virtually identical for most cases as shown in Fig. 5 that will be described below under Likelihood Inference.

For easy reference, the color coding of horizontal lines in (A) and (B) corresponds to the colors of the likelihood functions displayed in panels (C) and (D). Each likelihood at λ* = *c* in panel (C) is obtained by cross-sectioning the full likelihood in (A) at λ* = *c*.

Notice that the marginal likelihood *L*_2_ in panels (B) and (D) is quite flat over large ranges of *ψ* when λ* is held constant and that the largest values appears on either both or one side of *ψ* = 0 depending on the possible configurations of tables defined by the success total and sample sizes.

When the group sample sizes are equal, such as in Fig. 1 and Fig. 2, the surfaces of the marginal likelihood are completely symmetric with respect to *ψ* = 0 regardless of the magnitude of the sample size. This is because possible configurations of tables resulting in positive and negative MLEs for *ψ* are symmetrically distributed with respect to *ψ* = 0 and their numbers are equal (i.e. the number of values of $\tilde{\psi }$ above panel (B)). Thus, for equal sample sizes, the plots clearly show that the marginal likelihood given *y*_+_ is essentially flat, supporting our intuition that it gives no information about whether *ψ* is negative or positive.

On the other hand, when the sample sizes are unequal, as shown in Fig. 3 and Fig. 4, the marginal likelihood surface is asymmetric. The marginal likelihood in (B) and (D) shows whether a positive or negative value of *ψ* is more likely. In the extreme example of Fig. 4, the high bulk of the marginal likelihood in (B) appears on the positive axis of *ψ*, because the number of configurations of tables resulting in positive $\tilde{\psi }$ is much greater than that of negative $\tilde{\psi }$ (7 vs. 4). Especially, the marginal likelihood in (D) at $\lambda^{*}={\hat{\lambda }}^{*}=0.37$ tells us that a positive *ψ* is more likely than a negative *ψ*. Other than this potentially more likely direction, it tells nothing about the specific value of the MLE of *ψ*. Surprisingly, the MLE equals -2.2, which is the opposite sign, and far from the bulk, of the largest marginal likelihood values.

The likelihood surface plots from a variety of scenarios show very similar patterns (symmetric for balanced cases and only dependent upon possible table configurations regardless of the MLE of *ψ*), and suggests that the success total gives little information about *ψ*. Thus, knowing the total number of successes conveys very little information about *ψ*, agreeing with our intuition.

Many investigators have noted without clear explanation that the conditional MLE of *ψ* always lies between zero (the null) and the unconditional MLE [33, 37]. This is easily seen from our plots. The full likelihood is proportional to the product of the conditional and marginal likelihoods, and the height of the marginal likelihood at the MLE of λ* (thick blue line) under the peak of conditional likelihood (red curve) falls as *ψ* approaches zero. This shifts the value of the unconditional MLE away from the null value if the MLE is not zero.

Zhu and Reid [14] studied the information lost by using only the conditional likelihood based on the Fisher’s information matrix for *ψ* and *λ*. Their figures show that this loss is zero around *ψ* = 0 and gradually increases as *ψ* moves away from zero. Using the original parameterization of *ψ* and *λ*, as they do, we see an overall saddle like shape that is very similar to our marginal likelihood surface plots, with a saddle point at *ψ* = 0. Finally, we note that our discussion and conclusions in this section also apply to the original parameterization. We have used the orthogonal parameterization (*ψ*,λ*) in order to facilitate our intuition.

### Discretization of the sample space

It would seem from the previous discussion that most parties are in agreement, and that the nuisance parameter can be eliminated by simply conditioning on the total number of successes (because this approach sacrifices little, if any, relevant information about *ψ*). Why then does substantial disagreement remain?—i.e., why don’t we just use a version of Fisher’s ‘exact’ test based on the conditional likelihood? Or, put another way, why is Fisher’s exact test overly conservative when compared to Pearson’s *χ* ^2^ test, given that there is virtually no loss of information from conditioning? To solve this puzzle, we look to the sample space, upon which the p-value is based.

While conditioning on *y*_+_ changes the likelihood for *ψ* in only minor ways, it increases the discretization of the sample space. This, in turn, creates problems when the likelihood function is interpreted by reference to the sample space. The problem manifests itself in how the p-value should be computed. For example, consider again the data in Table 1. For these data, Table 3 shows the original sample space (i.e., without conditioning) while Table 4 shows the sample space with conditioning on *y*_+_. Without conditioning, the sample space consists of 11 × 11 = 121 discrete points from all combinations of *y*_1_ and *y*_2_. However, the sample space is dramatically reduced to only 7 points when conditioned on the observed success total. The p-value from Fisher’s exact test is calculated using the reduced sample space and this results in larger p-values compared with Pearson’s *χ* ^2^ test. This happens even though the conditional and unconditional likelihoods both provide virtually equivalent statistical evidence (i.e., their likelihoods are nearly identical) because there is essentially no information loss due to conditioning.

**Table 3:** The sample space for the data in Table 1 where *n*_1_ = *n*_2_ = 10 without conditioning: combinations of *y*_1_ and *y*_2_ yield 11 × 11 = 121 possible configurations of tables. The sample space with conditioning on the observed success total is in bold face.

	*y*_2_											
		0	**1**	2	3	4	5	6	7	8	9	10
*y*_1_	0	(0, 0)	(0, 1)	(0, 2)	(0, 3)	(0, 4)	(0, 5)	(**0**, **6**)	(0, 7)	(0, 8)	(0, 9)	(0, 10)
	1	(1, 0)	(1, 1)	(1, 2)	(1, 3)	(1, 4)	(**1**, **5**)	(1, 6)	(1, 7)	(1, 8)	(1, 9)	(1, 10)
	2	(2, 0)	(2, 1)	(2, 2)	(2, 3)	(**2**, **4**)	(2, 5)	(2, 6)	(2, 7)	(2, 8)	(2, 9)	(2, 10)
	3	(3, 0)	(3, 1)	(3, 2)	(**3**, **3**)	(3, 4)	(3, 5)	(3, 6)	(3, 7)	(3, 8)	(3, 9)	(3, 10)
	4	(4, 0)	(4, 1)	(**4**, **2**)	(4, 3)	(4, 4)	(4, 5)	(4, 6)	(4, 7)	(4, 8)	(4, 9)	(4, 10)
	5	(5, 0)	(**5**, **1**)	(5, 2)	(5, 3)	(5, 4)	(5, 5)	(5, 6)	(5, 7)	(5, 8)	(5, 9)	(5, 10)
	6	(**6**, **0**)	(6, 1)	(6, 2)	(6, 3)	(6, 4)	(6, 5)	(6, 6)	(6, 7)	(6, 8)	(6, 9)	(6, 10)
	7	(7, 0)	(7, 1)	(7, 2)	(7, 3)	(7, 4)	(7, 5)	(7, 6)	(7, 7)	(7, 8)	(7, 9)	(7, 10)
	8	(8, 0)	(8, 1)	(8, 2)	(8, 3)	(8, 4)	(8, 5)	(8, 6)	(8, 7)	(8, 8)	(8, 9)	(8, 10)
	9	(9, 0)	(9, 1)	(9, 2)	(9, 3)	(9, 4)	(9, 5)	(9, 6)	(9, 7)	(9, 8)	(9, 9)	(9, 10)
	10	(10, 0)	(10, 1)	(10, 2)	(10, 3)	(10, 4)	(10, 5)	(10, 6)	(10, 7)	(10, 8)	(10, 9)	(10, 10)

**Table 4:** The sample space for the data in Table 1 where *n*_1_ = *n*_2_ = 10 with conditioning on the success total: there are only 7 possible table configurations. The observed *y*_1_ and *y*_2_ are in bold face.

*y*_1_	0	**1**	2	3	4	5	6
*y*_2_	6	**5**	4	3	2	1	0
*y*_+_	6	**6**	6	6	6	6	6

So we see that the dilemma of differing p-values is really caused by the change in the sample space and not by something more substantial. The justification for using a p-value to represent the strength of evidence against the null hypothesis is given by the Repeated Sampling Principle [29]. It says that inferences should be based on the long-run frequency properties of the statistical procedure that generated the data. However, there is a conceptual problem with the Repeated Sampling Principle: the frequency properties of the statistical procedure that generate the data are confused with the strength of the statistical evidence in a given set of data. We can see the confusion here as virtually identical likelihood functions yield different p-values because of changes in the sample space.

The Likelihood Paradigm avoids this conceptual defect by using separate mathematical quantities for (1) the strength of evidence, (2) the probability that a study design will yield misleading evidence, and (3) the probability that the observed evidence is misleading [38, 39]. In short, one simply examines the likelihood function to see what the data say about *ψ* and since conditioning on *y*_+_ changes the likelihood in only minor ways, the issue of further sample space discretization is avoided. The frequency characteristics of this approach are naturally dependent on the sample space [(2) above], but this quantity is now clearly distinct from how an observed likelihood function is interpreted (i.e., how strong the observed evidence is in the data). We will briefly expand on this theme in the next section.

### Modes of inference for 2 × 2 tables

While the controversy surrounding the best modeling choice for a 2 × 2 table appears to rely on the specification of the margins, the antecedent is really a question of specifying a working model that is computable (i.e., free of nuisance parameters). The principles heretofore discussed inform the choice of a working likelihood, but often the decision is not transparent (c.f., [5, 8, 25–27]). However, the manner in which the likelihood function is interpreted—e.g., directly or with referenced to the sample space—brings additional diversity to this debate that should not be ignored.

#### Significance testing (Frequentist inference)

The merits—and lack thereof—of significance testing are well understood [40–43]. Without revisiting this debate, we note a few points relevant to this paper’s focus.

P-values for 2 × 2 tables are based on the probability under the null hypotheses of obtaining tables as or less likely than the one observed. Unless the sample size is large with a moderate numbers of successes, there are only a limited number of configurations of an observed 2 × 2 table (see, for example, Table 4). As a result, the sample space upon which the p-value is based can become highly discretized [23]. The effective lack of a continuum for the strength of evidence in the data results in a conservatism that is truly problematic: the probability of falsely rejecting the null hypothesis is less than the nominal level. That is, the observed p-value is greater than it ought to be, if this statistic is to be interpreted as a consistent measure of strength of evidence against the null hypotheses. Fisher’s exact test is conservative and has been sharply criticized for this reason [9, 22]. Unconditional tests, such as the chi-square test, mitigate the discreteness of the sample space since there are more hypothetical tables based on unobserved success totals. However, this approach does not completely resolve the problem.

There has been considerable discussion of this controversy in the statistical literature. We raise just a few points here. One approach to smoothing p-values is to randomize the test outcome after the results are observed [19]. Of course this grossly violates the Conditionality Principle. Several authors [20–22, 44] propose or advocate for versions of the *mid*-p-value. Brazzale *et al.*[45] showed that the higher order approximation to the p-value is very similar to the *mid*-p-value. In terms of good frequency properties, a good alternative to be the p-value can be found by back-calculating from an appropriate conditional likelihood ratio (LR) test statistic using a *χ* ^2^ approximation:

$$\begin{array}{c} -2\log\left(L\left(0;y_{1}|y_{+}\right)/L\left(\hat{\psi };y_{1}|y_{+}\right)\right)∼\chi_{1}^{2}. \end{array}$$ (9)

This is different from the conventional Pearson’s *χ* ^2^ test in that it is a monotonic function of the conditional LR, free of reference to the original sample space, and therefore inferentially consistent with a likelihood ratio (see also the discussion of LR interval estimation for a binomial proportion by Brown *et al.*[46]). While the p-value based on this LR test statistic is inferentially more consistent than other p-values, we argue in the next section that strength of evidence can best be measured by the likelihood directly. The LR-based p-value should only be used when editors or referees require a p-value.

#### Likelihood inference

The Law of Likelihood is an axiom for interpreting the strength of statistical evidence in a given set of observations under a given model [42, 47]. The likelihood ratio measures the strength of evidence for one simple hypothesis versus another. The likelihood function displays the evidence over the entire parameter space, which is why proponents of likelihood inference say looking at the likelihood function is sufficient to see ‘what the data say’. Alternatively, likelihood support intervals are reported. A 1/*k* support interval is the set of all parameter values that are consistent with the data at a likelihood ratio level of *k*. These intervals are analogous to the confidence intervals (CIs) of Frequentists or the credible intervals of Bayesians. See Royall [42] and Blume [39] for an introduction to this approach and for applications.

The Likelihood Principle—which says that if two sets of data yield proportional likelihood functions then these data sets provide equivalent instances of statistical evidence—is a direct consequence of the Law of Likelihood. As such, we forgo an in-depth discussion and make only two points: First, the Likelihood Principle follows from the Sufficiency and Conditionality Principles [48–50]. Secondly, the Likelihood Principle is often misunderstood and is only properly evaluated in the context of a well defined evidential framework [38].

While it is straightforward to apply the Law of Likelihood in situations where the likelihood function is indexed by a single parameter of interest, situations where the likelihood function is indexed by several parameters (often a parameter of interest and nuisance parameters) are more complicated because these nuisance parameters must be removed. It should be noted, however, that modern likelihood inference handles nuisance parameters quite readily, even in some cases where the statistical model changes as additional observations are taken [39, 42, 51, 52]. The key idea is that a working likelihood should closely mimic the behavior of the ‘true’ likelihood function in terms of how often it will yield misleading evidence. The primary tools for accomplishing this include conditional, marginal, integrated, profile, and modified versions of profile likelihoods [36, 45, 53–57].

##### Presentation of evidence and examples

For illustration, the standardized likelihood plots (i.e., likelihoods standardized to their maximum value) for the examples already displayed in Fig. 1, Fig. 2, Fig. 3 and Fig. 4 are presented together in Fig. 5 which shows the 1/6.8 (upper), 1/8 (middle) and 1/32 (lower) likelihood support intervals (SIs) along with the MLE, $\hat{\psi }$. The SI for *k* = 6.8 corresponds to the nominal (i.e., not adjusted) Frequentist 95% CI under a normal model [39, 42]. The standardized conditional and modified profile likelihoods [53] are indistinguishable, which is expected since the modified profile likelihood approximates the conditional likelihood when it is appropriate [58]. The modified profile likelihood of [53] is proposed to adjust uncertainty in the profile likelihood or to “correct” bad behaviors of the profile likelihood. Hence, the modified profile likelihood has better inferential properties than the ordinary profile likelihood [53, 59, 60], especially when many nuisance parameters need to be profiled out. For those cases, the MLE could be biased or the interval estimate could be too narrow [61].

We use Example 2 in Fig. 5 (the data in Table 1) as an example to illustrates how one might display the strength of evidence, from a likelihood perspective [42] in a 2 × 2 table. The conditional likelihood has MLE $\hat{\psi }=-2.08$, which is at least 8 times better supported over values outside of the 1/8 interval [-5.3, 0.07]. The null value, *ψ* = 0, lies within this interval meaning the data are generally consistent with this hypothesis. For comparison, note that the surrogate normal (1/6.8) SI [-5.14, -0.01] barely excludes *ψ* = 0, and hence the p-value based on LR test statistic is less than 0.05 as shown in Table 2. The likelihood ratio comparing the MLE $\hat{\psi }=-2.08$ to *ψ* = 0 is 6.89. Overall, the likelihoods represent only moderate evidence that Treatment 1 is superior to Treatment 2. Note that the modified profile and conditional likelihoods are indistinguishable as discussed above even with an extremely small and unbalanced example of data. The presentation of evidence for 2 × 2 tables using a standardized likelihood plot can be easily implemented using an R package *ProfileLikelihood*[62].

#### Bayesian inference

The Likelihood Principle also plays a central role in Bayesian inference since the likelihood function is the key component describing the evidence in the data. Bayesian methods provide the tools for accessing our degree of belief about parameters. Suppose that our interest is in characterizing how our beliefs change in the light of the evidence after observing data. Examining the posterior distribution typically does this. However, if the point is to quantify the evidence in the data, we need to be careful in using the posterior density for this purpose as it reflects the combination of the prior information and the evidence in the data.

It is true that the posterior density is driven by the likelihood function and will wash-out any prior information if the sample size is large enough. However, when the sample sizes and the number of successes in 2 × 2 tables are small, the posterior distributions can be sensitive to the priors, even with flat or noninformative priors. See Howard [63] for an example of Bayesian analysis for 2 × 2 tables, where results were quite sensitive to different priors.

There have been efforts to find Bayes procedures that have good frequency properties using an appropriate choice of priors such as a noninformative prior or a matching prior [64]. The Bayesian literature regarding this topic has been growing, but is largely beyond the scope of this paper. We would note that Brown *et al.*[46] showed that a Bayesian interval for a binomial proportion with the Jeffreys’ prior had good frequency properties.

#### Comparison of Frequentist, Bayesian, and Likelihood approaches

We performed a small scale simulation study to explore the similarity between the likelihood methods and compare them with those of Frequentist tests such as Fisher’s exact and Pearson’ *χ* ^2^ tests, and the Bayesian methods with two different noninformative priors (Jeffreys’ and uniform priors). This simulation study is not comprehensive (a comprehensive one would be beyond the scope of this paper). However, since we are most concerned about the Type I error rate relating to the criticism on conservatism of Fisher’s exact test, we examined several null scenarios with small sample size with balanced and unbalanced cases, and calculated the false positive rates. This simulation study and the results are described in S2 Appendix. As shown, a Bayesian interval for the log odds ratio with Jeffreys’ reference prior tended to be closer to the one from the conditional likelihood and had good frequency properties. We leave extensive simulation studies for future study.

## Conclusions

Two-by-two contingency tables provide fertile ground for examining the foundational principles of our everyday statistical practice. The controversy surrounding Fisher’s exact test for 2 × 2 tables is rooted in the (often hidden) foundational principles we use to make statistical inferences. The Sufficiency and Conditionality Principles play an important role here as they provide a basis for the specification of a working likelihood and the elimination of nuisance parameters.

Fisher’s exact test is based on the observation that the total number of successes is essentially an ancillary statistic and we have confirmed that virtually no information is lost by conditioning on this statistic. As a consequence, Fisher’s intuition that his test should be conditioned on the marginal success total is appropriate. Unfortunately, conditioning on the marginal success total can create a highly discretized sample space. This makes Fisher’s exact test p-value too conservative, and it is non-trivial to perform inferences within the Frequentist paradigm. On the other hand, inferential methods that directly interpret the conditional likelihood perform well and are less affected by the discrete sample space. If forced to choose among p-values for 2 × 2 tables, the one associated with the conditional likelihood ratio test, Equation (9), performs better than most. This p-value has the virtue of being consistent with statistics derived for normally distributed data; it implies the same strength of evidence against the null hypothesis as that for a normally distributed statistic with an identical likelihood ratio. In general, interval based methods—CIs, credible intervals, likelihood support intervals—are preferable to p-values although CIs do not resolve the problem for Frequentists, because the CI coverage is subject to the same discretization issues [23].

## Supporting Information

S1 Appendix Denitions of the Law of Likelihood, and estimated, prole, and modied prole likelihoods. (PDF)

S2 Appendix Comparison of simulation results from Frequentist, Bayesian, and Likelihood approaches. (PDF)
