# Supplementary material for: Elucidating the Foundations of Statistical Inference with 2 x 2 Tables
Source: PLoS One. 2015 Apr 7;10(4):e0121263. doi: 10.1371/journal.pone.0121263 (PMC4388855; doi:10.1371/journal.pone.0121263)
Supplement: S1 Appendix — (PDF) [file pone.0121263.s001.pdf]

Appendix A. Definitions of the Law of Likelihood, and estimated, profile, and modified profile likelihoods

Law of Likelihood: If hypothesis A implies that the probability that a random variable X takes the value x is $p_A(x)$, while hypothesis B implies that the probability is $p_B(x)$, then the observation $X = x$ is evidence supporting A over B if and only if $p_A(x) > p_B(x)$, and the likelihood ratio, $p_A(x)/p_B(x)$, measures the strength of that evidence [1].

Estimated likelihood: Given the joint likelihood $L(\psi, \lambda)$, the estimated likelihood of ψ is:

$$L_E(\psi) = L(\psi, \hat{\lambda}),$$

where $\hat{\lambda}$ is the maximum likelihood estimates of λ. The estimated likelihood is also called a 'pseudo-likelihood' in [2].

Profile likelihood: Given the joint likelihood $L(\psi, \lambda)$, the profile likelihood of ψ is:

$$L_P(\psi) = \sup_{\lambda} L(\psi, \lambda),$$

where the supremum is with respect to λ and is performed at each value of ψ, which is held fixed.

Modified profile likelihood: Given the joint likelihood $L(\psi, \lambda)$ and the profile likelihood $L_P(\psi)$, the modified profile likelihood of ψ is:

$$L_M(\psi) = L_P(\psi) \left| j_{\lambda\lambda}(\psi, \hat{\lambda}_\psi) \right|^{-\frac{1}{2}} \left| \frac{\partial \hat{\lambda}_\psi}{\partial \hat{\lambda}} \right|^{-1} = L_P(\psi) \frac{\left| j_{\lambda\lambda}(\psi, \hat{\lambda}_\psi) \right|^{\frac{1}{2}}}{\left| l_{\lambda;\hat{\lambda}}(\psi, \hat{\lambda}_\psi) \right|},$$

where $\hat{\lambda}_\psi$ are the maximum likelihood estimates of λ at a fixed value of ψ,

$\hat{\lambda}$ are the maximum likelihood estimates of λ,

$\hat{\psi}$ is the maximum likelihood estimate of ψ,

$$j_{\lambda\lambda}(\psi, \hat{\lambda}_\psi) = -\frac{\partial^2}{\partial \lambda^2} \, l(\psi, \hat{\lambda}_\psi; \hat{\psi}, \hat{\lambda}) \Big|_{\lambda = \hat{\lambda}_\psi} \quad \text{and}$$

$$l_{\lambda;\hat{\lambda}}(\psi, \hat{\lambda}_\psi) = \frac{\partial^2}{\partial \lambda \hat{\lambda}} \, l(\psi, \hat{\lambda}_\psi; \hat{\psi}, \hat{\lambda}) \Big|_{\lambda = \hat{\lambda}_\psi}.$$

References

1. Hacking I. Logic of statistical inference. New York: Cambridge University Press; 1965.
2. Gong G, Samaniego FJ. Pseudo maximum likelihood estimation: theory and applications. The Annals of Statistics. 1981;9(4):861–869.
