# Supplementary material for: Elucidating the Foundations of Statistical Inference with 2 x 2 Tables
Source: PLoS One. 2015 Apr 7;10(4):e0121263. doi: 10.1371/journal.pone.0121263 (PMC4388855; doi:10.1371/journal.pone.0121263)
Supplement: S2 Appendix — (PDF) [file pone.0121263.s002.pdf]

Appendix B. Comparison of simulation results from Frequentist, Bayesian, and Likelihood approaches

We performed simulation studies to examine the similarity between the likelihood methods, and also to compare them with those of Frequentist methods such as Fisher's exact and Pearson χ^2 tests, and the Bayesian methods with two different noninformative priors (Jeffreys' and uniform priors). We simulated 1000 replicates for each scenario and present the results from four of the null scenarios with different sample sizes. We excluded some replicates if $y_1 = 0$ or $y_2 = 0$ (empty cell) from our simulation since the maximum likelihood estimate (MLE) cannot be estimated for these outcomes (i.e., the likelihood is unbounded). We estimated the false positive rate (i.e., the probability of being rejected when the null is true) by calculating the number of replicates being rejected divided by the total number of replicates excluding the ones with empty cells (i.e., $y_1 = 0$ or $y_2 = 0$). This probability converges to the Type I error rate as the proportion of empty cells goes to zero. To compare with Frequentist methods, the LR test statistic, $T = -2\log\left(L(0; y_1|y_+)/L(\hat{\psi}; y_1|y_+)\right)$, was calculated. The null was rejected if $T > \chi^2_{1,1-\alpha}$ at $\alpha = 0.05$, assuming that it approximately follows a chi-square distribution with the degree of freedom of 1 (χ^2_1) under the null. The false positive rate for the Bayesian methods were also calculated based on the posterior distribution of ψ, $p(\psi|y_1, y_2)$, which was obtained from the posterior samples of $p(\pi_1, \pi_2|y_1, y_2)$ with uniform or Jeffreys' priors for π_1 and π_2. The null was rejected if $p(\psi > 0|y_1, y_2) < 0.025$ or $p(\psi < 0|y_1, y_2) < 0.025$ to be comparable with the Frequentist approach.

The comparison of the false positive rates between Frequentist, the Bayesian and the likelihood approaches is shown in Table 0.1. First, notice that the false positive rates are exactly the same for the conditional and modified profile likelihoods due to the similarity of the LRs. The profile likelihoods behave almost the same as the other likelihoods except for the very small differences in a couple of scenarios.

For the Bayesian methods as well as Fisher's exact and Pearson χ^2 tests, the false positive rates were also calculated with all replicates, including the ones with empty cells, presented in parentheses. Depending on the choice of priors, the posterior distributions varied substantially, especially for data with small samples and sparse cells. With the small sample size, there is not much information in the data so that the likelihood may be outweighed by the prior. Interestingly, the Bayesian approach with the uniform prior gave almost the same false positive rate as the Pearson χ^2 test. On the other hand, the Bayesian approach with Jeffreys' reference prior resulted in a rate that was very similar to the conditional and modified profile likelihoods.

We found that overall the likelihood methods are less affected by the excessively conservative properties of the Pearson χ^2 test and Fisher's exact test for small samples due to discreteness, while preserving the false positive rate quite well.

Table 0.1. Comparison of simulation results from Frequentist, Bayesian, and likelihood approaches. The 1000 replicates were simulated for each scenario, and the false positive rates were calculated by the number of replicates rejected at $\alpha = 0.05$ divided by the total number of replicates excluding ones which generated empty cells (i.e., $y_1 = 0$ or $y_2 = 0$). The proportion of empty cell is presented for each scenario. For Fisher's exact test, Pearson χ^2 and Bayesian methods, the false positive rates were also calculated with all replicates including ones with empty cells, presented in parenthesis. The likelihood ratio (LR) statistic, $T = -2\log\Big(L(0; y_1|y_+)/L(\hat{\psi}; y_1|y_+)\Big)$, was calculated for each likelihood method, where $\hat{\psi}$ is the maximum likelihood estimate (MLE) of ψ. The null was rejected if $T > \chi^2_{1,1-\alpha}$. For the Bayesian method, the false positive rate was calculated based on the posterior distribution of ψ, $p(\psi|y_1, y_2)$. The null was rejected if $p(\psi > 0|y_1, y_2) < 0.025$ or $p(\psi < 0|y_1, y_2) < 0.025$. Jeffreys' and uniform priors were used to obtain the posterior distributions.

		false positive rate			
		$n_1 = n_2 = 10$ $p_1 = p_2 = 0.2$	$n_1 = 10; n_2 = 20$ $p_1 = p_2 = 0.2$	$n_1 = n_2 = 20$ $p_1 = p_2 = 0.2$	$n_1 = 20; n_2 = 30$ $p_1 = p_2 = 0.2$
	empty cell (proportion)	0.218	0.123	0.018	0.009
Frequentist	Fisher's exact	0.001	0.021	0.020	0.032
	(with empty cell)	(0.007)	(0.021)	(0.023)	(0.034)
	Pearson χ^2	0.008	0.029	0.049	0.039
	(with empty cell)	(0.037)	(0.041)	(0.058)	(0.045)
Bayesian	Uniform prior	0.008	0.029	0.049	0.039
	(with empty cell)	(0.037)	(0.036)	(0.058)	(0.045)
	Jeffreys' prior	0.028	0.033	0.049	0.046
	(with empty cell)	(0.089)	(0.054)	(0.061)	(0.053)
LR (χ^2_1)					
Likelihood	Conditional	0.028	0.033	0.049	0.046
	Profile	0.028	0.033	0.049	0.048
	Mod. Profile	0.028	0.033	0.049	0.046
